# The Experience of International Students: Biographical Narratives and Identities

**DOI:** 10.1007/s12115-023-00809-0

**Published:** 2023-02-13

**Authors:** Pierluca Birindelli

**Affiliations:** 1 University of Helsinki, Helsinki, Finland; 2 International Studies Institute, Florence, Italy; 3 Gonzaga University, Florence, Italy

**Keywords:** International students’ mobility, Young adult identity, Biographical narrative, Autoethnography, Cosmopolitan

## Abstract

This article presents the findings of a qualitative and comparative study on the cultural experience of international students in North and South Europe. I employ a narrative approach and the focus of the research revolves around the autoethnographies of 25 international students in Helsinki and 25 in Florence. The narratives were prompted by in-depth interviews following a template divided into the three phases of travel conceived as a rite of passage: departure–preliminal, transition–liminal, arrival–postliminal. To explore the meaning of geographical mobility in the lives of these young people, I sketched a series of self-identity types connected to mobility experiences: the *Fated*, whose biographical premises are all pushing-pulling toward the status of international student; the *Academic*, who is fascinated by the idea of becoming a worldly intellectual and sees the PhD as a natural step; the *Globetrotter*, whose mobility is an end in itself: the goal is the next city-country; the *Explorer*, who is abroad looking for new cultural challenges, with a genuine desire to discover and understand specific places and people; the *Runaway*, who feels like a stranger at home and is escaping abroad for political or existential reasons. I believe that the interpretation of international students’ sense of self-identity can be fruitfully achieved through the narrative path I have constructed (or a similar one).

## Introduction

Over recent decades, studying abroad has increased vastly to become an institutionalized practice. International student mobility has expanded constantly over the past 20 years. In 2019, 6.1 million tertiary students worldwide had traversed a national border, more than doubling the 2007 figure (OECD 2021, 215). Years 2020–2021 will probably represent a watershed due to the coronavirus pandemic. It remains to be seen whether this is a turning point toward the decline of international student mobility or a temporary pause. In this study, the key question is not a quantitative one. It is not about the “how much” of the trend, but about “what” and “who.” Here I explore what will decline or resurge, who these international students are in biographical and narrative terms, and how the mobility experience impacts the way they imagine their future lives (Cuzzocrea and Mandich 2016).

Transnational and global higher education mobility is an impressive social and cultural phenomenon that has been, and still is, accompanied by two cultural knowledge gaps influencing each other: the scarcity of popular public narratives (such as books, movies, or documentaries) and of academic narrative-biographical studies. Because of this double and interdependent cultural void, I believe there is further scope for interpreting this study area in depth.

Secondary analysis of non-scholarly material revealed that it is very hard to find a book or a movie that represents the individual and collective meaning of traveling, living, and studying in another country. Here I mean a complete story, a narrative that introduces the protagonists at home in their familiar surroundings, portraying the sociocultural background along with the trigger factors leading to the decision to go elsewhere, and then the experience abroad, and how it affects the students’ life path and self-identity. To the best of my knowledge, the only story representing the “Euro-Cosmopolitan” student is still the 2002 movie *L’auberge Espagnole*.

Even within the academic domain, beyond quantitative socio-demographic data there is little qualitative empirical material for a deeper understanding of students’ overall experience from an authentically narrative-biographical and comparative slant.^1^ Moreover, existing studies^2^ tend to suffer from separation into distinct disciplines (sociology, social and cultural anthropology, communication studies, education, social psychology, cultural studies) and thematic fields (youth, human development, mobility studies, cultural globalization and cosmopolitanism, education). In addition, in most studies, the protagonist (the international student) is conceived and represented as a mere “agent”: we know very little—and sometimes nothing—of his/her biographical past. To find out what young people are really getting out of higher education mobility, we need to hear their stories and explore the implications of the educational travel within the broader context of their lives: past, present, and future.

This qualitative and comparative research addresses, among others, the question: Is studying abroad fostering cultural openness through real opportunities to meet the Other in the flesh or does it support a more aesthetic “touristic gaze” (Urry and Larsen 2011)? In other words, can international students’ narratives disclose other core meanings beyond the implicit/explicit, instrumental-expressive significance of cultural self-empowerment abroad (Papatsiba 2005) to meet the challenges of a globalized world? I believe so.

This research led me to formulate another core, and potentially foundational, meaning for the studying-abroad experience that I conceptualize as *existential*. If the possibility of imagining oneself “elsewhere” is a fruit of late modernity and cultural globalization, an imaginative consequence for the construction of individual identity is a sort of quest for “one’s place in the world”: a personal promised land. If “developing a cosmopolitan identity is at the core of discourses on educational travels” (Huang 2021, 4), this study revealed how studying abroad can be considered a dual transitional passage toward adulthood and global citizenship (Birindelli 2018). In short: a training camp to become cosmopolitan (Hannerz 1990, 2005),^3^ albeit without exactly knowing what “cosmopolitan student” means.

The objective of the research project *The Cultural Experience of International Students* is to interpret the biographical meanings attributed by a group of 50 international students to their educational, cultural, and overall life experience abroad, in Finland (North Europe) and Italy (South Europe). The study employs a cultural and narrative-biographical approach developed over the years (Birindelli 2014, 2022), and its overall purpose is to reconstruct students’ narrative self-identity “at home” in their past, during their stay abroad (present), and in their attempt to imagine themselves either in the host country, back at home, or elsewhere (future). Hence, in this study I encouraged and collected partial autobiographies-autoethnographies—autoethnography being the description of self as seen within another culture (Ellis and Bochner 2000). The collected stories have an authentic narrative and biographical structure: incipit–ruit–exit; past–present–future. To the best of my knowledge, this kind of systematic study has never been carried out for international students.

In this research, “identity” is understood as “narrative identity.” Narrative identity (Ricoeur 1984, 1985, 1988; Bruner 1990; Burke and Stets 2009) is always a retrospective interpretation of the past and an anticipation of the future. Identity is a process made up of the relations that the individual—along with the intersubjective inside-outside group recognition—establishes, through memory, between the different and shifting perceptions of oneself in relation to the Other, and to the wider sense of belonging to a (national, regional, transnational, global) collective identity (Birindelli 2022, 14).

## Method and Conceptual Framework

### Developing a Novel Method

Starting from September 2016, I found and recruited 25 international students at the University of Helsinki (Finland, representing Northern Europe) and 25 at the University of Florence (Italy, representing Southern Europe).^4^ I was able to contact the participants of the research in Finland with the support of supervisor Keijo Rahkonen, at the time head of the Department of Social Sciences and Vice-Dean for international affairs. Thanks to his help, I was also able to contact international students’ associations.^5^ In Italy, this part of the research process was more difficult because students’ associations—and student life in general—do not exist, for either international or local students:^6^ another finding that will be interesting to analyze in the future. In Florence, I was supported by the pro-rectors of the University of Florence at the time, Marco Bindi and Giorgia Giovannetti, and by other directors of International Master Programs, especially Valeria Fargion. Although Florence can be considered an international student city, I struggled to find participants for the research. In order to preserve a comparability criterion, they had to be international master students in a public university. In Florence, the majority of international students are undergraduates—European Erasmus students, US abroad programs (Birindelli 2020)—or PhD students, for instance enrolled at the European University Institute.

Regarding the sampling method, qualitative inquiry typically focuses in depth on relatively small, purposively selected samples. Unlike random sampling, with logic derived from statistical probability theory, the logic and power of purposive sampling lies in selecting information-rich cases to study in depth, from which one can learn a great deal about issues of central importance to the inquiry (Denzin and Lincoln 2000). I might add that my research project gleaned knowledge from individuals with particular expertise. Expert sampling is particularly useful where there is a lack of empirical evidence in an area, which is the case of my investigation.

I carried out the fieldwork during the academic year 2016–2017, and in two follow-up phases in 2020 and 2021 when I started to share interpretations in a Facebook group discussion. Thus, I studied the international students over a 5-year timespan. I am unaware of any similar longitudinal study ever having been carried out for international students, so that it represents a pioneering contribution (in the field).

At the start of the study, after some informal conversations with undergraduate students, I decided that the participants in the research would be Master students. I saw the Master students as the “older brothers and sisters” of the younger undergraduates I had already researched in the past; being older, they would be able to reconstruct their stories with a greater degree of reflexivity. Also, I imagined that I might meet young people who had previous exchange experience and, so to speak, insisted on going abroad. This anticipation was correct and became a significant dimension of the study. In the attempt to come up with narrative identity types, I first created the *Veteran*, a student who went through at least three levels of studying-abroad experiences: high school, undergraduate, and master. I later dropped this type because almost all the international students I met in this study can be considered “veterans.”

Overall, I was able to achieve a balance in terms of age (the average was 26 at the time of the final draft of the autoethnography) and gender, and to get students from all inhabited continents involved, while for the area of study/discipline I had to settle for the available International Masters in the two universities. In this study, I chose to research students from different nations instead of focusing and comparing just a few nationalities—such as in the quantitative study (survey) of Finn et al. (2022). This is because I was interested in exploring the international students’ sense of belonging to a “cosmopolitan group” and concentrating on similarities-differences of their experience abroad grounded in their biographical narratives rather than national or regional culture.

The phases of the fieldwork were broken down as follows.

1. In-depth interviews (approximately 1 h and a half)
  These were based on a narrative template.
  The full transcription of the interview, with my preliminary interpretation of some key points and/or questions, was given to the participant who revised, integrated, changed, and deleted at will. This transcription prompted the autobiographic-autoethnographic reflection.
2. Autoethnography (average of 15 pages, single spaced)
  The autoethnography was based on the same template as the interview. The participant was also free to develop other topics and/or to decide to develop certain themes of the template in greater or lesser depth, since it was his/her story.

The overall design of the research project was constructed to foster participants’ involvement as active subjects rather than passive objects. The possibility of reading transcripts and preliminary interpretations in every single phase made the participants feel that they were not only actors, but also to a degree scriptwriters and directors of the research process, thus laying the foundations for a cooperative enterprise based on trust.

### Liminality and the Narrative Template: a Heuristic Conceptual Overlay

The life stories of young people constitute the backbone of this research itinerary. All biographical accounts and other research steps were guided by a *narrative template*. The template is divided into sections addressing the three basic phases of travel (departure–transition–arrival) creating a heuristic overlay with the “three phase architecture” at the heart of the study: narrative structure (incipit–ruit–exit); existential time (past–present–future); rites of passage (preliminal–liminal–postliminal); human development (young–young adult–adult); sense of belonging to a collectivity (national–European–cosmopolitan). This overlapping (Table 1) constitutes a novelty both in the method and in the theoretical construction of the research itinerary.

**Table 1:** Heuristic overlay in the narrative template

1.	*Phases of travel*	Departure	Transition-passage	Arrival
2.	*Existential time*	Past	Present	Future
3.	*Narrative structure*	Incipit	Ruit	Exit
4.	*Rites of passage*	Preliminal	Liminal	Postliminal
5.	*Human development*	Young	Young adult	Adult
6.	*Belonging to a collectivity*	National	European	Cosmopolitan

While conceptual dimensions such as *phases of travel*, *existential time*, and *narrative structure* do not require further explanation, the inextricable connection between rites of passage (liminality), human development transition, and sense of belonging to a collectivity calls for clarification.

Young people today face a dual human development transitional passage: (1) in the dimension of individual and generational identity (youth–adulthood); (2) in the sphere of collective identity and sense of belonging (national–global). For those who were born (or moved to live) in the old continent, it becomes a triple liminal phase: becoming adults, citizens of a globalized world, and Europeans (Birindelli 2018).

As regards the *sense of belonging to a collectivity*, the traditional *human development transition* from youth to adulthood also needs to be conceived in a transnational and global manner: space and “spatial reflexivity” (Cairns 2014; Cairns et al. 2018) need to be incorporated into the study of young people. Yet this interpretative approach should not simplistically dismiss the role of the country-state in both its concrete structural impact in peoples’ lives—all passports worldwide, for instance, are still nation-based—and cultural reverberations: the country of origin, although redefined, remains a powerful source of symbolic meanings molding collective and individual identities. The outside and “inside-out” narrative of a collective identity is as important as the cross-boundary one (Birindelli 2019). We can in fact transcend a boundary only by recognizing its existence. Additionally, by analyzing international students’ narratives, I have realized how the key criterion defining their international status is precisely being inter‐national(s). The cosmopolitan status of a student is inevitably conferred by the marker of joining, somewhere abroad, a group of people coming from different countries. The cosmopolitan game simply ends if you take away the reference to the country of origin—tricky, isn’t it? There can be no universality without particularity and vice versa: “Cosmopolitanism, in short, is empty without its cosmos” (Harvey 2000, 554).

Regarding the *liminal dimension*, analysis reveals that studying abroad can constitute a rite of passage (Van Gennep 1909/1960): a liminal and transitional space-time toward adulthood, Europeanism, and/or cosmopolitanism (Birindelli 2018). Indeed, when international students leave “home” (their comfort zone, usual living area, educational environment, etc.) and travel to a new place, they must adapt to a new ecological system with its social and cultural scenery. It is crucial to emphasize the liminal dimension (Turner 1969, 1977) for several reasons. The institutional endorsement given by global academia, family, friends, etc. (society at large we might say)—channeled mainly through internet and social media—constructs a framework of codified practices, procedures, and symbolic meanings for studying abroad. The life transition toward what we can call “cosmopolitan adulthood”^7^ takes place within the travel path designed by the departure from the homeland, the transition to the host land, and the definitive or temporary return home. The liminal phase clearly takes place during the sojourn abroad, and the cosmopolitan status is achieved only after a series of highly codified steps, such as passing exams and earning the degree. When the young person (on the brink of adulthood in our case, thus young adult) completes this ritual-cultural path, family, friends, and the academic community (both at home and abroad) should recognize the new sociocultural status of international student. Analysis of the collected autoethnographies tells us that this is not happening. The international student’s postliminal stage remains unclear and consequently, since it is a narrative, even the preliminal and liminal stages become hazy.

In his essays, Victor Turner introduces an interesting term, *liminoid*: the “successor of the liminal in complex large-scale societies, where individuality and optation in art have in theory supplanted collective and obligatory ritual performances” (Turner 1987, 29). Therefore, liminoid manifestations can challenge the broader social structure, a kind of cultural critique of the *status quo*. Can the studying-abroad experience be equated with Turner’s “liminoid”? I doubt it. I have found no evidence that studying-abroad challenges the *status quo*: quite the opposite.

We can instead preserve the quasi-liminal meaning of liminoid experiences, in the sense that they are optional and do not lead to the resolution of a personal crisis or a change in status. Liminal events are ritual forms of cultural performance and involve society as a whole, whereas liminoid experiences are essentially transitional and the individual can choose to participate in or ignore them (Turner 1974). Furthermore, we should not lose sight of how the studying-abroad season is nested in the life passage from youth to adulthood. And however prolonged, fragmented, culturally diverse, global, etc., it remains an adulthood realized through a job and other concrete conquests. At the end of the 2002 movie *L’auberge Espagnole*, although the protagonist Xavier has, through his father, the chance of a good job in a ministry, he instead decides to pursue his childhood dream and become a writer. His first book is, of course, about himself and his postgraduate Erasmus experience in Barcelona. Through this narrative escamotage, the script unintentionally suggests that the protagonist gets stuck in his liminal time. The narratives of studying abroad suggest something akin to what Szakolczai (2017) called “permanent liminality.” Under static, petrified conditions “change in the sense of creativity, innovation, and adventure, in a word, ‘liminality,’ is most welcome,” whereas permanent liminality, to use a Foucauldian term is intolerable: “It generates a sense of *stasis*, meaninglessness; the more things change, the more they stay the same” (Szakolczai 2017, 244).

### The Lack of Public Narratives: Scripts Without a Story

Interpretation of the international students’ autoethnographies—the core of this study—was supplemented with secondary sources, adding layers of information, and using one type of data to validate or refine others (data triangulation). Analysis of all the qualitative empirical material took place within a broad framework of scholarly and non-scholarly inter- and multidisciplinary sources focusing the themes directly or indirectly related to the research. Therefore, along with the fieldwork, I collected and/or analyzed quantitative and qualitative data on young people studying abroad; scholarly articles, essays, and monographs; and non-scholarly material (books, movies, social media and travel blogs, documentaries, advertisements, music, tourist guides, audio-visual data, news media, documents, archives, etc.).

Within the *departure–preliminal* section of the in-depth interview, I also asked the participants about the kind of images and stories they had of the host city/country and the experience of studying abroad in general, and about the media sources of such representations, specifying that they could be anything. Here I was essentially trying to reconstruct international students’ imaginary of the host city-country and of the studying-abroad experience by searching for “cultural objects” (Griswold 1994) that might have shaped their expectations. Subsequently, I concentrated my analysis of the collected autoethnographies on movies because of their power to shape a narrative and mold evocative representation of the overall experience abroad—extensive interpretation of this section can be found in Birindelli (2021).

The only movie mentioned (twice) representing the story of an international student was *L’auberge Espagnole—Pot Luck* or *The Spanish Apartment* in English.^8^ I then conducted the secondary analysis of movies at national (Finnish, Italian) and international level concentrating on three key criteria: (1) portrayal of the story of the student-protagonist with a slight “coming of age” narrative approach; (2) pertaining at least to the comedy-drama-romance’s genre—I discarded movies such as Lizzie McGuire’s and all “rom-com” (romantic comedies); (3) high level of international diffusion (10 or more national markets reached) and having a European city as movie setting. The analysis revealed only one possibility: *The Spanish Apartment*.

The interpretation of the collected autoethnographies and of secondary scholarly and non-scholarly sources connected with studying abroad reveals the absence of a clear-cut narrative of what it means to be an international student. We can find a series of related images, but not sufficient to constitute a leading narrative for students’ life experiences in North Europe, South Europe, and Europe in general or elsewhere. It is possible to glimpse a vague cosmopolitan narrative, constructed on a global scale by different actors and institutions, upholding the generic validity of studying abroad for both instrumental and expressive reasons, and as an institutionalized rite of passage toward adulthood and global citizenship. However, it remains unclear what is the prize, the elixir, the treasure, or the lesson (Campbell 1968; Propp 1928/1968) to be gained from the special “studying-abroad world” and what the young person is going to do with it in adult life. Consequently, the liminal state of studying abroad can be reconceptualized as either “liminoid” or “limbic.” And this ritual interpretation is consistent with young people’s endlessly prolonged mobile trajectories, at times leading nowhere from an economic and socio-demographic viewpoint (Cuzzocrea and Cairns 2020).

My analysis leads me to interpret the (quasi-) ritual of studying abroad as a *script without a story*, i.e., a structured story, such as a book or a movie. If this is the case, the implicit and preconscious meaning of studying abroad grows enormously. The young person’s self-story lacks a center of narrative gravity, leaving the student-protagonist alone both in acting and telling his/her epic. As a result, the overall myth-ritual is sabotaged, and even the recognition of the new cosmopolitan status by the community (institutions, family, peers, etc.) becomes blurry. It is one thing to enact on the basis of a story, and another to enact in the absence of a story.

As stated at the beginning of this article, in 2019, 6.1 million tertiary students worldwide had traversed a national border. However, this vast social and cultural process gave birth to very few popular narratives apart from *L’auberge Espagnole*, where the protagonist Xavier, a 24-something French Erasmus postgraduate in Barcelona, shares the apartment with other students from England, Belgium, Spain, Italy, Germany, and Denmark.

The film’s exclusive focus on young European Erasmus students already underlines the aims and limitations of what is supposedly a broad cultural and educational exchange. The emphasis on learning about “other” national cultures to achieve a more integrated European union quickly dissolves when *the students abandon any interest in local culture, history or politics to focus instead on their own sexual and emotional rites of passage*. (Ezra and Sánchez 2005, 137, emphasis mine)

In the collected autoethnographies, we can assume the presence of word of mouth, oral stories, where students share accounts of their experience abroad with friends and family. And we can also see many experiences abroad shared through pictures posted on Facebook or Instagram. There are also written stories in social media posts and some students keep a travel blog with more descriptive and systematic updates of their life abroad. On the institutional side, universities have dedicated websites describing their academic programs along with information on the social and cultural life in the host city and country such as “10 reasons to study at the University of Helsinki.”^9^ In addition, each program advertises itself by posting videos with professors and past students as testimonials (ambassadors) along with other videos targeting international students.

Yet, the analysis of the collected autoethnographies left me with a feeling of narrative vacuum. There is some sort of apparent self-evidence about going abroad being the “right thing to do” in expressive and instrumental terms—expanding one’s cultural horizons, enriching one’s CV—but the narrative void creates a major obstacle to finding a plausible answer to one of the core questions of this study: “What is the meaning of studying abroad?”

The narrative account is in fact the primary and most potent interpretative and cognitive tool that human beings, as socially and culturally situated subjects, can utilize to make sense of their life experiences (Bruner 1991). The time dimension, the self-narration, and the self/hetero-recognition dynamic are pivotal to the concept of identity: lived experience has a pre-narrative quality and personal life is “an activity and a passion in search of a narrative” (Ricoeur 1991, 29). Following Levinas, Ricoeur (1992, 187) argues that there is “no self without another who summons it to responsibility.” The Other who performs this action substantially expresses a judgment about the individual, placing him/her within a system of categories: gender, age, cultural belonging, social status, education, work, etc. Identification by others features different degrees of stability, depending on the extent to which one’s social profiles can be defined, and is subject to constant review. Self-identity is therefore constructed and reconstructed in the dialog with this internalized Other, yet this inter-intrapersonal narrative necessarily fishes in the sea of public narratives fixed in books or movies (Birindelli 2022).

In this study, the lack of intersubjective, common scripts leaves the international students alone in the attempt to make sense of their experiences abroad. If identity is a process, a construction of—and through—the individual and collective memory framework (Halbwachs 1980), what happens when the collective, public box is empty, or filled with scattered fragments of stories? The international student seems to epitomize the typical late-modern subject who must find new ways of implementing a reflection on himself that appears multi-faceted, complex, and, in some respects, solitary (Birindelli 2022).

There is a hiatus between the cosmopolitan promise made by academic institutions and the intellectual, cultural means given to the international student. Moreover, in the untold story of studying abroad even Self-Other identity dynamics becomes blurred: which Other? Which Self? What happens, then, to the hyperbolically claimed goal of going abroad to expand one’s horizons through the experience of a different Other? Are we talking about a local Other? Or is it “another like oneself”: an international student from a different country, but who is essentially—and reassuringly—remarkably similar to yourself?

## Findings and Discussion: Key Revelations from the Students’ Narratives

My aim here is not to give a complete and exhaustive analysis of all the collected narratives but to disclose “snippets”; otherwise, the explanation of the research itinerary, of the qualitative tool, and of certain core interpretations would be too speculative, hence unclear and misleading.

The *departure–preliminal* section reconstructs the social and cultural background against which the decision to study and live abroad took place. The *transition–liminal* section of the template addresses the actual academic and overall life experience abroad, while the *arrival–postliminal* section probes a bond with a human being in the host culture and with a place that became familiar during the stay. In this section, I also encouraged the students to reflect on their immediate prospects: returning home, staying in the host country, or moving somewhere else.

The three autoethnographical phases of travel were introduced at the beginning of the in-depth interview by the *collective identity pre-preliminal*: here I asked the participants to give short descriptive accounts of the country of origin, hometown, host city and country, and north/south Europe, and to define the word “cosmopolitan.” The autoethnography ends with the final free interpretation of *self-identity and the experience abroad* and with a *sociographic appendix* where participants in the research provided some simple but important information, such as their social class, parents’ jobs and education, and family members’ experiences of transnational mobility.

### Collective Identity Pre-preliminal

For obvious reasons, territorial reflection is an ice-breaking and stimulating way to start the interview with international students. The question “where do you come from?” can be imagined as the entry ticket for participating in the abroad game: the lack of a clear original geographical boundary would not allow its transcendence.

IT (m, Middle East, Hel) thinks his country of origin is “complicated, difficult” and the people are “stubborn, aggressive, impatient, loud.” Hometown clearly represents the past, and toward the end of the autoethnography (*arrival–postliminal*)—being a narrative study, the researcher needs to move up and down the entire autoethnography to make a thicker sense of ideas expressed in one part or the other—he writes “I really would like to stay here, but I can imagine myself also moving to other places, but not back to ***.” While his home country is “boring, the past” and the people are “arrogant, cold, elitist,” Finland is “a new home, village, cozy, fun” and Finns are “different, nice, friendly, inward.”

In the attempt to portray students’ orientations toward their particular home-worlds and the wider cosmopolitan elsewhere, I sketched out a series of self-identity types connected to mobility experiences. That said, obviously none of the students falls completely within the analytical boundaries of a single type: their self-narrative simply reveals (to me) various characteristics of one or more types.

IT reveals a self-identity sketch partially encapsulated by the *Runaway* narrative type: someone who is escaping abroad for political or existential reasons (they feel strangers at home). IT also shows characteristics of the *Academic* type: he is intrigued by the idea of becoming a worldly intellectual, “Professionally, I want to do PhD, academic life”; in the next 10 years, he sees himself “In the beginning of an academic career, as a junior lecturer, with a young family.”

IT gives positive meanings to the word cosmopolitan: “An inspiring, optimistic vision.” International students in Helsinki give either positive, neutral, or negative meanings to “cosmopolitan.” Neutral meanings relate to the idea of metropolis, while negative connotations range from inequality to snobbish lifestyle. Positive meanings are usually associated, both in Helsinki and Florence, with open-mindedness, tolerance, and appreciation of diversity. A negative idea of “cosmopolitan” is absent for the Florentine group, where the neutral meanings prevail—with several students unable to give a connotation to the word: “Politics maybe… but I do not know what it means”; “A magazine? A drink? I heard the word, but I never thought about what it means.” The meanings given to “cosmopolitan” are apparently ambivalent, divergent, and far from being shared.

### The Departure–Preliminal

Here I reconnect the meanings of studying abroad to the participants’ biography, moving beyond the sociological representation of a subject without a past, an “agent without a story.” Students’ biographical past is often, if not always, neglected in this field of study, whereas an authentically narrative approach is required to interpret students’ overall experience abroad, which cannot be confined to a singular biographical timeframe.

The autobiographical accounts in the departure section reveal a sort of *push-pull identity dynamic* triggering the desire to travel, live, and study somewhere else, away from home. For the *Fated*, all the biographical premises push-pull toward the status of international student. As one student writes “I almost had no choice but to study abroad” (NS, m, East Asia, Hel). NS’s parents met while the father was studying abroad during his bachelor’s degree. Even his mother studied abroad in her youth to perfect a foreign language. Several relatives on the father’s side had a studying-abroad experience, and his two sisters live in the USA after a study abroad period. NS’s decision to study in Helsinki grew totally within his family culture: “I guess that this kind of experience was not alien for me. I guess they [parents] also wanted me to do the same thing, to study abroad.” And there is a convergence also between family and country culture: “Because generally speaking if you get a foreign degree in *** you will be seen as more employable on the job market.” Furthermore, even his hometown played a role in the decision to go abroad: “I am from the capital. There are a lot of people with a rich background, rich upbringing. So, the experience of living, of studying abroad is quite a common thing.”

NS’s high school had an exchange program with a bordering country—“Already at a very young age I was socialized to the option of studying abroad”—and at his university, they support students financially to go abroad. NS had his first studying-abroad experience as an undergraduate in 2012 (6 months in a European country), so he is a studying-abroad “veteran”—like almost all the participants in the study. Sentimental life is no exception in NS’s biography; his ex-girlfriend was an international master student in a north European country. NS concludes “I guess it was not a fresh idea for me to study abroad”: the *Fated*.

Narrative traits of the *Runaway*, the *Academic*, and the *Fated* types emerged already in the departure–preliminal section, and sometimes, as for IT, even in the pre-preliminal section. Other types were revealed later in the autoethnographies. I will restrict myself to synthetic passages for each of the remaining types; however, I beg you to indulge me on the *Runaway*, since this type brought to light an unexplored meaning for the international student. ZW (m, Central Europe, Hel) comes up with a definition of the *Runaway* international student.

I met many international students here. I got the feeling they came here because they did not like their life back at home. It’s not that they said this out loud, I had the feeling that they were *running away from something*. Either because they are not happy with their home country, with the political situation, or maybe it’s because of the personal situation.

For the *Globetrotter*, being mobile is an end in itself: the goal is the next city-country. HN (f, North Europe, Hel) tells us that she has already visited 50 countries. She also did a quite extraordinary exchange program in East Asia that represented a watershed moment: “When I was there I discovered that I really like traveling, living in another city.”

Before I went there, I never really considered leaving ***. I had kind of a plan, and a boyfriend, and a career path. I was going to be a psychologist. Then I went to *** and everything changed. I decided I don’t want to do psychology and I don’t want to stay in ***. I *want to travel a lot and live in different places*.

HN is not a *Fated*; her family culture cannot be considered highly mobile. However, her mother has always been encouraging: “She said I know you need to do this, you need to go. I guess she did some traveling when she was in her twenties that a lot of people would not have done.”

The *Explorer* had previous experiences abroad that do not fall within the social and cultural perimeter of the “studying-abroad world.” ND (m, Oceania, Hel) worked overseas for long periods in places with a totally different climate from his home country. ND cultivates a strong desire to discover and understand places and people, always looking for new cultural challenges, both with other international people and with the locals, showing the capacity to reach out for the indigenous, thus, in his own words, “bursting the international students’ bubble” away from the “mobility capsule” (Czerska-Shaw and Krzaklewska 2021). Compared to the *Globetrotter*, the *Explorer* decides to stay longer in “a” foreign country, and she/he is not interested in visiting as many countries as possible. ND genuinely wants to understand, absorb, and integrate with this particular culture, the Finnish one. He did his undergraduate exchange in another Finnish city, and even then he did not live only in the international students’ bubble: “There are many reasons why it was a good experience, but I think one of the main good reasons is because I made friendship with Finns.”

The *Lover* is abroad because of the partner. ZN (f, Central Europe, Hel) was an undergraduate exchange student in Helsinki. She met her current Finnish boyfriend and now she has a clear idea why she is in Helsinki.

*I am here because of my boyfriend*. Originally, also because I loved to be surrounded by international people. There is a big gap between my Erasmus experience and my master’s degree experience. In the end, I stayed because of my boyfriend.

The *Worker* is studying abroad for clearly instrumental reasons. ZH (m, South Asia) is in Helsinki “to study and have better job opportunities” and HV (f, South Asia) “I’m studying *** [a top-level master program] at the University of Helsinki and my whole purpose of being here is education.” Contrary to what is commonly believed, studying abroad at Master level is not necessarily closely connected with the acquisition of skills in view of a job. After some exploratory in-depth narrative interviews, I decided to stimulate the students by asking them to answer the following straightforward question: “Why am I in Helsinki/Florence?” Only 13 students out of 50 wrote that they were abroad for academic reasons.

NV (m, Central Europe, Flo) has a dual cultural view shaped by his belonging to an ethnic and cultural minority. Even NV’s narrative shows the identity traits of the *Explorer*. He is willing to face cultural challenges and he is able to interact with both international and local people. He is, of course, a study abroad veteran, and the decision to come to Florence was shaped by previous experiences that, in his case too (as for ND), do not entirely fall within the studying-abroad boundary: a typical narrative characteristic of the *Explorer*. NV, in fact, did 1 year of European Voluntary Service in a small town in Finland. Furthermore, his traveling is strongly motivated by an intellectual curiosity rooted in the field of study: he is abroad for a specific reason (typical of the explorer) that transcends generic instrumental ends (embellish the CV, job, etc.) or expressive goals (expand one’s cultural horizons, growing as a cosmopolitan person, etc.). In Florence, NV is autonomously studying Machiavelli and doing some *sui generis* ethnographic work, visiting places connected to Machiavelli’s biography. He is more attracted by the substance of intellectual life rather than having the *Academic*’s fascination with the role. NV’s drive to travel and live abroad is certainly shaped by his family background; his grandparents emigrated to *** in the 1960s. However, the most striking aspect of NV’s story is that he is the only international student in the study who convincingly identifies his social class as “middle-lower”—lower in my interpretation.

I was raised in a marginalized region of ***, it was full of migrants from Turkey, Poland, Russia. I felt rich in a cultural way. That made me curious about other countries. Growing up in that neighborhood in *** sparked the motivation why I am living abroad.

Thus, besides *Explorer*, I also identify NV with the narrative type *Maverick*: someone who comes from a middle-lower/lower-class family and does not share the highest common denominator of international students: an upper, upper-middle, or middle-class family with significant cultural capital.

I was having difficulty in interpreting the Florence group using only the narrative types I created for the international students in Helsinki, so I created an extra type only for Florence: the *Tourist*. The length of the stay (2 or more years) and their advanced student’s status were not enough to sabotage the hermeneutic potential of the tourist narrative type. RH (f, EurAsia) writes: “The idea was to combine studying and traveling. I’ve been a tourist in Italy and I liked the country.” And she adds: “Actually, Florence is not a good city to live in. It’s good to see museums, art, history, but it is not a city for normal everyday life.”

### Transition–Liminal

The *transition–liminal* section explores different academic and life experiences abroad (city life, housing, friends, education, interaction with locals, social life, etc.). Here, and in the postliminal section, I will concentrate on emblematic autoethnographic passages dealing with *social life* abroad and the *interaction-experience with locals*.

QS (m, North America, Hel) reveals traits of the *Explorer*: he is abroad for a specific reason and his past experiences do not fall entirely within the studying-abroad boundary. QS is on track to become a pastor and he chose the Master in Religion, Conflict, and Dialogue. He had a privileged experience because he has personal interests and skills fundamental to his identity that allowed him to establish contacts with locals, thus escaping, even momentarily, the international students’ bubble. It was his biography that allowed him to open “a” door to local culture, rather than institutional assistance from the University of Helsinki or students’ associations.

Analysis of the autoethnographies reveals that you cannot experience the host culture holistically and vaguely. Access to local people takes shape only through an active attitude; it entails specific social skills, and “doing” things: performing (sports, religion, volunteering, a part-time job, etc.) rather than consuming or passively participating in social events of all sorts. QS goes to church every week and “through that I made connections with people that actually live in my neighborhood.” This is a fundamental point; through the group of churchgoers, he can meet locals and get involved in their social life. It is “one kind” of Finnish people—believers who go to church—but it is an important breach in the cosmopolitan bubble toward a slice of the particular local culture. A social competence that is part of the subject’s biography and self-identity becomes “a” key (not a passe-partout) to enter “a” door of the host society leading to some specific meaningful social space and group of people.

Later in the autoethnography, QS writes:

One of the families I got to know at church invited me to their house for Easter dinner… I left my bike there for the summer. I am probably a *little odd* in that respect, I was able to develop relationships *outside the academy, outside of the international students’ group*.

Sociologists tend to converge in defining cultural cosmopolitanism as an orientation of openness to foreign others and cultures, inspired by Hannerz’s seminal study of cosmopolitanism as “an orientation, a willingness to engage with the Other” as well as “the aspect of a state of readiness, a *personal ability* to make one’s way into other cultures … a built-up skill in maneuvering more or less expertly with a particular system of meanings and meaningful forms” (Hannerz 1990, 237–251, my emphasis).

My study confirms that this “ability to make one’s way into other cultures” is indeed personal. In this study, it seems that cosmopolitan orientation is provoked less by Hannerz’s “intellectual and aesthetic stance of openness toward divergent cultural experiences” (1990, 239) and more by the social skills that allow the international student to open certain cultural doors and engage with real people. Only through social activity can cosmopolitanism take on “a” shape. And, in an apparent paradox, cosmopolitanism is achieved only when students break free of the cosmopolitan study abroad bubble. This is what can be expressed as *social cosmopolitanism* enabling genuine and concrete contact with people of another culture, different age, social class, etc.

This cultural ability is personal and cannot be activated or facilitated by the host university. Students’ associations in Helsinki have the important function of connecting international students with each other or with local students who appear remarkably similar to them. They coined a sociological label for this kind of Finnish student; as IT (m, Middle East) writes: “Most of my social life was and still is with international students. Now I have some Finnish friends. But I call them ‘International Finns’, they join our events and act as if they were foreigners here. We use the formula *Internationally Minded Students*.”

That said, the importance of international students’ associations as a key socializing function is undeniable, making it possible to build the community of cosmopolitan students abroad. Although associations cannot guarantee an entry into local social life, they do bestow a sense of identity and community abroad and a form of engagement that is not just recreational or consumerist. Based on this study, the absence of such associations in Florence is seen as a minus. KS (m, East Asia) writes: “One of the strange things here is that there are no students’ associations. I’ve been in a student organization for all my academic life back in Asia. Student life basically does not exist here.” OY (f, West Asia) adds: “That I know, there are no students’ associations, students’ life is not organized. That is a problem, I could not find a community to join.”

The presence of numerous Erasmus groups with leisure connotations (party, happy hours, discos, touristic trips) underscores the recreational nature of social life in Florence. International education does not stop at the gates of the university campus, and this side of the social and cultural experience abroad also needs to be addressed, especially in a tourist city like Florence.

### Arrival–Postliminal

Part of the *arrival–postliminal* section consisted in portraying a bond with a human being in the host culture and with a place that became familiar during the stay. Here too I concentrate on autoethnographic passages focused on social life in general, and the interaction-experience with locals. I also explore the perception of the host city, attempting to see how the urban space might have shaped social relations and the connection between the experienced social and cultural space and international students’ future.

For the *human bond*, most of the students in Helsinki indicated a classmate or the group of fellow students abroad. Besides QS, who created a strong connection with the family he met at the local church, most of the bonds are with people within international academic confines; I believe this is quite normal and understandable. There is also, however, another recurrent strong form of relationship: the boyfriend/girlfriend. In these cases, the partner is always a Finn and represents a disclosure of the local world. Otherwise, it is rare to find a student who creates a strong bond with a local.

Regarding the *non-human bond*, Helsinki is considered a functional and livable city, and most of the international students truly appreciate this feature. IN (m, South America) feels a connection with the city of Helsinki because “It’s a peaceful and safe city. I can walk in the streets without worrying that I can be robbed or mugged or something.” The narrative passages dedicated to the city of Helsinki reveal a general appreciation of the harmony between the city and the natural world, along with the cultural vivacity, good public transport, and its overall functionality. However, what emerges from the narratives is a generic reference to “places by the sea” and—besides the obvious familiarity developed with the neighborhood where they live or university buildings and communal areas—none of the students developed a strong form of emotional attachment to a specific place, either because of its beauty or its comforting or reassuring function.

International students in Florence somehow managed to create more *human bonds* with local people. Beyond the typical creation of strong forms of relationships within the in-group of studying-abroad students, somehow the international students bubble seems less impermeable in Italy. Moreover, bonds do not follow the sentimental relationship path as in Helsinki, where several students had an indigenous partner. KS (m, East Asia), besides other students from his home country, “made a bond with Italian friends, we study together.” XO (m, South Asia) was able to establish a strong connection with “Four people… They are there for me anytime. A guy from ***, a guy from ***, and ***, she is Italian.” KG (m, Africa) made “a strong bond with three Italian guys. It’s a strong bond that will last forever.”

Friendship with locals involves mostly classmates and other Italians presented by them. It is apparently easier to become friends with an Italian than with a Finnish classmate. Because of the absence of students’ associations in Italy, and the touristic tradition of the city, at the beginning of this study I imagined exactly the opposite. A possible alternative interpretation in line with the findings is that, while international students’ associations in Helsinki (promoted and fostered by the university) represent a plus in many senses, at the same time they strengthen the spontaneous and conventional ties with other international students and contribute to foster the construction of a sort of enclave. Conversely, the “institutionally abandoned” international student in Florence is almost forced to establish contacts with locals, while Italian students feel almost compelled to reach out to their international peers.

The *non-human bond* with the city reveals another antithetical cultural dynamic between Florence and Helsinki. WW (f, EurAsia) writes “I have a strong bond with the city. I love Florence. Even if I am alone, I would still want to live here. In Florence I do not need people, the city is all I need”; MU (m, South America) echoes “I feel connected. I love Florence. I really do love this city.” The group of international students fell in love with the city of Florence. ON (f, East Europe) comes up with a generalization: “All international students have one thing in common: they all love the city of Florence. That is the first reason mentioned by any international student I met,” and later in the autoethnography adds: “Nobody I met mentions studying as a reason to come to Florence. The only reason is the love for the city.”

This brings us to the next narrative prompt regarding reflection on the immediate prospects: returning home, staying in the host country, moving somewhere else. Students in Florence would stay for the beauty of the city, the lifestyle, etc., but do not see Florence (Italy in general) as a suitable place to concretely construct their future life. DA (m, Africa) writes: “After the master I will go back home. I would like to stay here but as for job opportunities is better to go back home. Staying here with a master’s degree will not make a difference.”

Florence seems to represent a liminal moment painted with the colors of a vacation from real life. Some students are ready to make compromises to stay, which I interpret as the desire to prolong the vacation. One student mentions Milan as a possibility, a few mention the language barrier, but in my interpretation the main obstacle is that they do not see Italy as a stage for an ordinary everyday life where work has a pivotal role. Italy is the perfect country for an extraordinary life: a vacation. This probably explains why I created an extra type only for Florence: the *Tourist*.

Only one student indicates the charm of the city of Helsinki (or Finland/Finnish lifestyle) as the main reason for being abroad, whereas almost half the Florence students fell in love with the city, country, lifestyle, etc. The passion for Florence (Tuscany, Italy) has the contours of a confirmed expectation, while the enthusiasm for Helsinki (Finland) seems to develop during the stay, without any kind of premise molded by representations and images found in the media in the broad sense (movies, documentaries, books, internet, social media, etc.). However, international students saw Helsinki and Finland as somewhere they could build their real life. Several students wanted to remain in Finland after the Master but foresaw obstacles in terms not of skilled job opportunities, as with Florence, but of language: “There are not that many jobs for English speakers in Finland. So far I haven’t got any job I have applied for… But I haven’t closed the door to staying in Helsinki just yet” (PW, f, South Asia).

Beyond job opportunities, sentimental relations are obviously decisive for the *Lover* type: “My boyfriend’s father has a big company here. Here I have almost zero job chances. I feel I have to decide between my boyfriend and my career” (ZN, f, Central Europe). *Runaways* rule out the possibility of going back home. IT (m, Middle East) writes “I really would like to stay here, but I can imagine myself also moving to other places, but not back to ***”, and adds that “I want to do PhD, academic life.” As noted, IT’s autoethnography also reveals the traits of the *Academic* type. Although in qualitative studies numbers do not make much sociological sense, it is interesting to observe how in the Helsinki group one international student out of three wants to pursue an academic career.

The *Academic* is not a secondary narrative type in this study. Participants in the research spent the past 10 years in international education at different levels. Academe probably constitutes an important, if not the most important, “province of meaning” in their paramount reality of everyday life (Schutz 1962/2012). The master program does not signify a bridge toward a professional working life, but a required step to do a PhD and remain within academe, which is a big part of the “real life” they have experienced so far.

During the in-depth interviews, I asked students to imagine *the recognition of the self-identity status change* brought about by the life experience abroad. Interestingly, the majority (both in Helsinki and Florence) imagine such recognitions within their private sphere: family and friends. Rather than institutional others or generalized others (Mead 1934), it is a recognition from significant others (Sullivan 1953) in the personal sphere: “My mom will tell me that she is really proud of me, of where I am and what I am doing” (PW, f, South Asia, Hel); “My family and my boyfriend. Even my friends from university. They appreciate what I am doing abroad” (OY, f, West Asia, Flo).

In the last 20 years or so, universities worldwide have been deeply engaged in some sort of internationalization process. So, why are international students, the protagonists of this process, not duly celebrated? There are generic references to the importance attributed to an international Master degree in the home country, but the scarcity of more formalized recognition of academic achievements and change of status is evident. Interpreting studying abroad as a rite of passage, a postliminal form of recognition within the academic community is clearly missing, tending to sustain the *liminoid* or *limbic* characteristic of studying abroad. The absence of celebratory occasions attended together by the academy, family, and friends—the community as a whole—casts uncertainty and ambiguity on the newly acquired status of cosmopolitan student. Again: what is it? What’s the story?

Even this final section of international students’ autoethnographies reinforces the already stressed interpretation of a “rite without a story”: in this case, what is missing is “the end” of a season of life, chapter, etc. The absence of any ritualistic forms of recognition—either by the host university abroad or the home university—is striking when compared with the vast studying-abroad phenomenon: a core, if not “the” core, administrative objective of universities worldwide.

The value of studying abroad sometimes seems confined to the international students’ group: “International students that are living like me, that are in the same existential situation give value to what I am doing here” (MU, m, South America, Flo). As for the outgroup, studying abroad sometimes has a meaning that stands between generic instrumentality and distinction practice, and the latter is far from unusual in this study.

If I go back to *** it will be very *cool*. Makes me stand out from the crowd. If I go somewhere in Europe it might be cool as well. Because many people know that a Finnish degree, at the University of Helsinki is very good. (EP, f, EurAsia)

Why throw out “cool” as a possible interpretation for the popularity of studying abroad? Isn’t it also cool to be cosmopolitan rather than local or parochial? More than 20 years ago, Bauman (1998, 2) wrote that “mobility climbs to the rank of the uppermost among the coveted values” and that “the freedom to move, perpetually a scarce and unequally distributed commodity, fast becomes the main stratifying factor of our late-modern or postmodern times.”

I finally pushed the international students of this study to stretch their existential imagination into the *future, 10 years later*—where? doing what? etc.—trying to guess the meaning of the studying-abroad experience in that envisioned future, hence exploring a possible shift of significance during their imagined adulthood. This final part helps us to rebalance the academic and scholarly vision of international students as the spearhead of the cosmopolitanizing process, a vision that is sometimes overly stretched, to the point of seeing them as political actors or cultural brokers instead of simply young people on the brink of adulthood. What we find in the last section of their autoethnographies is often a family, a job, a nice house, and some good friends. Ten years from now, “I hope that at least I have a house. A family. And a job that I like. Hopefully in Finland and in a nice area of Helsinki” (QK, m, South Europe). The projection into the future features the desire to have a normal, good life, stressing the overall self-identity growth of the studying-abroad stage on the way to adulthood.

I will look back and be very proud of what I’ve done. I learned a lot here. I was not a very open person when meeting new people, but I have changed and become more sociable with others. I have broadened my circle. It’s my overall experience as a person. (SZ, f, Africa, Hel)

If you stay at home you do not develop your potential, you need to go abroad. There is so much to see and experience and you need to do that in person, otherwise you remain with just a poor, superficial and most of the time wrong idea with media images of the world, of other people. (OY, f, West Asia, Flo)

It is *self-growth*. The cultural challenges and the experience of the cultural Other—albeit sometimes another that looks a bit too much like you—make international students grow as young adults. Since they are not social scientists or political activists, why should it be otherwise?

## Conclusions: Overcoming Hermeneutic Obstacles and Contribution to Literature in the Field

In this article, I have presented an overview of a comparative and qualitative study of the academic, social, and cultural experience of international master students. By reconstructing the temporal bridge between the phase of life abroad and students’ lifespan tout court, the narrative-biographical approach pursued fills a current knowledge gap within the multi-faceted interdisciplinary field studying youth, human and educational mobility, cultural globalization, and cosmopolitanism. In this research, the time abroad is conceived as a building block in students’ self-identity construction and as a stage of their human development: young, young adult, adult. By yoking together in a truly narrative guise time and geography—past-home, present-abroad, and expectations for the future either in the host country, back home, or elsewhere—I was able to interconnect time and space dimensions and overcome a knowledge gap even in this sense.

Narrative-biographical studies of this kind are rare, and this scarcity constitutes the *first* hermeneutic obstacle: the researcher has no solid scholarly and fieldwork props to lean on. It is only by reconstructing students’ stories that we can reach a broader and deeper understanding of the academic and overall experiences abroad within the concrete context of young people’s lives. Only a narrative-biographical study can bring to light hidden meanings and open hermeneutic itineraries that go beyond the international student mobility “axiom.” The current pandemic-induced pause calls for a questioning of the assumed significance of mobility and a reconceptualization of the mobility-immobility dyad in new conditions of “disimagined mobility,” where portraying a clear and attractive vision of a grounded transition to adulthood becomes problematic (Cairns et al. 2021).

The conceptual mesh and the method I propose, or a similar one, appears to be a viable interpretive path for acquiring a holistic understanding of this social and cultural phenomenon. The narrative template I created for this study is at once simple and comprehensive. It can certainly be improved. However, I believe that the basic narrative-biographical structure should be kept intact: it is impossible to reconstruct a reliable portrait of the impact of studying abroad on students’ life without a systematic reference to their past, present, and future. Since we are addressing mobility, I am also confident that the hermeneutic overlay with the phases of travel is valid—with analytical attention to ritual (liminal/liminoid/limbic) conceptual meanings—because the international students I met in this study are trying to travel in many senses: geographically, culturally, socially, and existentially.

Subsequently, I pointed out other interpretative hindrances. The vacuum of qualitative studies of this kind is surrounded by another narrative void: the absence of public and popular stories (mainly novels and movies) on studying abroad: the *second* obstacle. The lack of such tales undermines our capacity to reconstruct in a well-rounded fashion the traits of the protagonists of the studying-abroad story: the international students. My attempt to create narrative types such as the *Fated*, the *Explorer*, and the *Runaway* stems precisely from this awareness. Of course, it is an attempt that can be improved but at the same time I believe it cannot be easily dismissed. While waiting for the storyteller to write new poetic scripts for our heroes, we must try to reconstruct in a prosaic academic way the profile of the young person as a character in his/her life story.

There are two more major obstacles to this kind of research that have so far appeared only between the lines of this article. The *third* obstacle is easy to grasp: people out there are not eager to tell their life story to any passing researcher and write an autoethnography. Thus, it takes time, hard work, and reciprocal trust to carry out the fieldwork. Once you have succeeded in finding participants and conducting in-depth interviews, I see giving the full transcription back to participants as the only possible way to prompt the writing of a partial autoethnography. Afterwards, we can meet, and do focus groups and the like. But the gist of this kind of study lies in the transformation of an oral story (the in-depth interview) into a written story (the autoethnography), with the commented full transcription as a middle step.

The *fourth* obstacle is the required multidisciplinary approach. Once the researcher gains a sufficient knowledge to carry out fieldwork and interpret from different disciplinary angles—in my case sociology, social and cultural anthropology, psychology, and narrative studies—it is hard to publish the results in journals. Editors and peer reviewers will always ask you to be “on top” of each of the disciplines they are expert in. People doing this kind of fieldwork can be seen as “detectives” looking for clues and linking them together, after which they present their evidence to the academic theorists, whose role is to connect different disciplines into a new, understandable, whole.

As for some of the main studying-abroad meanings revealed by this narrative and autoethnographic research, I would synthetically point out the following. (1) The *existential significance* of studying abroad: rather than being instrumental (studying in view of a highly skilled job) or expressive (expanding one’s cultural horizons and alike), the experience abroad seems more the search for a sort of personal promised land within a culturally globalized world. (2) The scarcity or absence of *postliminal institutional forms of recognition* for the academic experience abroad: due to the vastity of international students’ mobility worldwide, one would expect more systematic rituals of cultural-structural recognition for young academic achievements. (3) The extreme relevance of personal skills grounded in the students’ biography that allows some of them to competently perform in a field (sports, religion, volunteering, a part-time job, etc.) giving access to a slice of the host culture (what I called *social cosmopolitanism*) rather than vaguely consuming the culture or passively participating in social events of all sorts. (4) The *uncertainty of the meanings attached to “cultural cosmopolitanism”* within the overall life experience abroad and the need for effective operationalization of the concept: the “internationally minded students” label that appears in students’ autoethnographies portrays a cosmopolitan group separated from the local Other.

I believe this study has made a contribution to refinement of the concept by tracing the contours of the “cosmopolitan student” on the brink of adulthood, or at least of the international students I met. And the students I met have their ideals, their dreams, their fears. Despite being highly mobile, they are all searching for their place in this world and, however you want to put it, this “niche” consists of a job, a house, a partner, and some good friends. Moving away from all of this would be a big sociological mistake.

Attempting to draw a synthetic conclusion from what I studied, my point is that “out there” in the stormy cosmopolitan sea what’s missing most of all is the image of a “good life,” a life worth living even far from the bright lights. I do not see how one can become cosmopolitan, a “good citizen” of a global world without stories of a “good life” that are not a mirage.

## Footnotes

1 Rare examples of qualitative research of this kind on international students are the following: Murphy-Lejeune (2003); Papatsiba (2005); Brooks and Waters (2010); Carlson (2011); Krzaklewska (2013); Cuzzocrea and Mandich (2016). Murphy-Lejeune’s 2003 study and approach is probably that which has most affinities with mine. However, her research consists of 60 interviews featuring precise questions exploring the researcher’s legitimate theory of the “stranger,” forms of adaptation to a new culture, and culture shock. My narrative template is simpler and does not influence or guide students’ self-narratives. I believe it can be more easily adapted by other researchers engaged in the field of international student mobility, studying abroad, or youth studies in general. From a conceptual perspective, and especially in interpreting the studying abroad experience within the lifecycle framework, this study has most affinities with those of Cuzzocrea and Cairns (2020) and Cuzzocrea and Krzaklewska (2022): I have discussed this article with these authors and I thank them in the Acknowledgments. Regarding the autoethnographic-autobiographic method, in most existing literature this tends to be the researcher’s personal account of the experience of living and working in contexts other than the country of origin (Daskalaki 2012; Daskalaki et al. 2016). An example of this method applied to studying abroad can be found in Nilemar and Brown (2019) where, adopting an autoethnographic approach, the author offers a first-person account of the past experience of being an international student in various countries. To the best of my knowledge, in available literature there is no study with 50 autoethnographies (or autobiographical narratives) written by international students from all inhabited continents.

2 On International Student Mobility (ISM) studies, see among others the following: Byram and Feng (Eds. 2006); Feyen and Krzaklewska (Eds. 2013); Dervin and Regis (Eds. 2015); Van Mol (2014); Cairns (2014); Cairns et al. (2018). On the biographical approach to youth transition, see Henderson et al. (2006).

3 A clear and foundational overview of the cosmopolitan scholarly debate can be found in the introduction to the book edited by Vertovec and Cohen (2002). Besides Hannerz, leading scholars who reactivated the debate upon cosmopolitanism in the sociological and anthropological field are, among others, respectively Beck (2006) and Appiah (2006). For an educational, philosophical, and historical perspectives, see Papastephanou (Ed. 2016). For a discussion of the cosmopolitan *bildung* of young people, see Cicchelli (2012), and for the cosmopolitan *habitus* of international students, see Igarashi and Saito (2014). Regarding cosmopolitanism as an empirical field of research in the social sciences, see Kendall et al. (2009). An extensive overview of the multi- and interdisciplinary cosmopolitan debate can be found in Delanty and Inglis (Eds. 2011), Delanty (2009, 2019), Skrbis and Woodward (2013), and Cicchelli and Mesure (2020).

4 In this article, I present the study and my interpretations in a narrative style. I believe this stylistic choice and storyline is more authentic and consistent with the kind of study I carried out, guided by a narrative template.

5 Mainly CISSI (https://blogs.helsinki.fi/internationalcissi/) and TSEMPPI (https://blogs.helsinki.fi/tsemppi-hy/).

6 In Florence, there are several students’ groups for finding accommodation or with a clear recreational connotation. They are mainly groups of Erasmus or post-Erasmus students with a Facebook or Instagram page. They organize trips, dinners, aperitifs, an disco-nights. None of these groups stems from or is linked to the University of Florence, and they do not carry out cultural activities stricto sensu.

7 On spatial mobility as a resource in the transition to adulthood, see among others Thomson and Taylor (2005), Camozzi (2022), and Grüning and Camozzi (2023).

8 This Franco-Spanish co-production was an international commercial success; the movie costs 5,300,000 euro to produce and, between its release in June 2003 and August, earned 18,732,000 euro in France and Spain alone (Ezra and Sánchez 2005, 137). At world level (11 national markets), the movie made $33,272,835 (BoxOfficeMojo, retrieved December 2022). This success led to the release of two sequels in which studying abroad was not the main theme: *Les Puppées Russes* (*Russian Dolls,* $23,727,301, 13 markets) and *Chinese Puzzle* (2013, $16,968,297, 24 markets).

9 In the webpage dedicated to international students: https://www.helsinki.fi/en/admissions/for-international-students.
